# The Effects of Hot Air and Microwave Drying on the Structural and Physicochemical Properties of Soluble Dietary Fiber from Sugar Beet Pulp

**DOI:** 10.3390/foods14193435

**Published:** 2025-10-07

**Authors:** Xinmeng Huang, Zunqi Zhang, Yuanpeng Li, Yuting Yang, Ailikemu Mulati, Dilireba Shataer, Jiayi Wang

**Affiliations:** College of Smart Agriculture (Research Institute), Xinjiang Key Laboratory of Biological Resources and Genetic Engineering, Xinjiang University, Urumqi 830046, China

**Keywords:** sugar beet pulp, soluble dietary fiber, hot air drying, microwave drying

## Abstract

Sugar beet pulp (SBP), a byproduct of the sugar industry, presents significant potential for enhancing economic benefits and promoting sustainable development through its comprehensive utilization. SBP is rich in fiber, with its soluble dietary fiber (SDF) constituting a high-value component. The initial step in the preparation of SDF involves the drying of fresh SBP. This study compares the effects of hot air and microwave drying on the composition, structure, and physicochemical properties of SDF in SBP. Technologies such as gel permeation chromatography, gas chromatography–mass spectrometry, Fourier transform infrared spectroscopy, scanning electron microscopy, and Zeta potential analysis were employed. Results indicated that microwave drying enhanced sugar components in SDF, reduced polysaccharide molecular weight, and formed a uniform and porous microstructure. This resulted in a higher Zeta potential (−24.76 mV) and increased water holding capacity (5.01 g/g). Hot air-dried samples preserved a more intact cell wall network, exhibiting higher swelling capacity (5.18 mL/g). The study demonstrated how both drying methods differentially regulated SDF quality from sugar beet pulp, suggesting that drying process selection should be based on specific application needs.

## 1. Introduction

Sugar beet pulp (*Beta vulgaris* L.), commonly known as sugar beet, is a vital crop in global sugar production systems. Following the industrial extraction of sucrose through mechanical pressing, the by-product, sugar beet pulp (SBP), constitutes approximately 70–80% of the initial beet biomass [1]. Due to its relatively low economic value, this lignocellulosic material is traditionally used as animal feed or discarded as waste. However, recent emphasis on sustainable resource valorization has spurred research aimed at optimizing the utilization pathways of SBP. This agro-industrial residue is notably rich in dietary fiber, with a typical composition of approximately 20% cellulose, 25% hemicellulose, 25% pectin, and minor proportions of protein, lignin, and ash content [2]. This unique biochemical profile positions SBP as a viable feedstock for producing high-quality dietary fiber. Although primarily used in animal nutrition, scientific studies have demonstrated that dietary fiber extracted from SBP exerts multiple beneficial effects on digestive physiology: it facilitates ion exchange processes, exhibits slow fermentation kinetics, ameliorates lactate-induced acidosis, enhances microbial degradation of crude fiber, and supports lipid metabolism through betaine-mediated pathways, which may partially fulfill methionine and choline requirements [3].

Dietary fiber (DF) is primarily classified into soluble dietary fiber (SDF) and insoluble dietary fiber (IDF). SDF is characterized by its notable health-promoting properties, including high solubility, substantial water- and oil-holding capacities, interfacial activity, and the ability to adsorb harmful substances [4,5]. A representative bioactive compound, ferulic acid, a natural polyphenol found in sugar beet SDF, acts as an antioxidant by neutralizing free radicals, thus serving as an essential functional component [6]. However, these bioactive constituents are often encapsulated within dense fibrous networks, resulting in limited bioaccessibility. Appropriate drying treatments can effectively alter the structural integrity of dietary fibers, facilitate the exposure of active sites, and enhance the release and absorption of bioactive compounds [7].

Drying is a pivotal unit operation in the processing of SBP. The high moisture content of fresh pulp hinders storage and transportation efficiency, often leading to nutritional degradation and a subsequent reduction in feed value. Consequently, the advancement of efficient and energy-conserving drying technologies is crucial for the comprehensive valorization of SBP. Various drying methods, including vacuum drying, freeze drying, and combined drying techniques, can be employed to process SBP. However, this study focuses on comparing microwave drying (MD) with hot air drying (HD). Although conventional HD is scalable and compatible with existing equipment, it is associated with high energy consumption, extended processing times, and low thermal efficiency [8]. Moreover, prolonged thermal treatment may negatively impact the functional properties of dietary fiber, such as pectin depolymerization and the occurrence of undesirable Maillard reactions, which collectively contribute to reduced bioactivity [9]. As an emerging heating technology, MD exhibits significant potential in the processing of agricultural products due to its high efficiency and energy-saving attributes. The two methods differ markedly in their heating principles: HD utilizes heat conduction and convection to transfer heat from the surface inward, whereas MD involves direct internal energy absorption, heating the material from within. This allows MD to transfer energy directly into the interior of the material. When processing an equivalent mass of SBP, the time required for MD is substantially less than that for HD [10]. The enhanced drying kinetics further contribute to reduced energy consumption and operational costs. Additionally, studies suggest that MD better preserves the nutritional profile and may induce structural modifications within the dietary fiber matrix, such as disruption of crystalline regions and exposure of hydrophilic functional groups, thereby enhancing both the content and functionality of SDF [11,12].

This study systematically investigates the effects of HD and MD on the compositional, structural, and functional characteristics of SDF extracted from SBP. The molecular weight distribution of SDF was determined using gel permeation chromatography (GPC), providing insights into degradation patterns induced by drying. Monosaccharide composition analysis was conducted via gas chromatography–mass spectrometry (GC-MS) to evaluate changes in glycosidic linkage integrity, degree of polymerization, and sugar composition. Fourier transform infrared spectroscopy (FT-IR) was employed to identify alterations in functional groups, such as esterification and deacetylation. Microstructural features, including surface morphology, aggregation behavior, and porosity, were visualized using scanning electron microscopy (SEM), while zeta potential measurements were applied to assess particle surface charge and its implications for colloidal stability and solubility. Finally, key physicochemical properties, including water holding capacity (WHC), oil holding capacity (OHC), and swelling capacity (SWC), were evaluated to determine the influence of drying methods on techno-functional performance. The effective extraction and utilization of dietary fiber from SBP may offer new opportunities for applications in the food and pharmaceutical industries [5]. This work provides a theoretical and technical foundation for understanding how drying conditions influence SDF quality and supports the rational selection of processing methods for value-added applications of sugar beet by-products.

## 2. Materials and Methods

### 2.1. Optimization of Drying Conditions and Extraction of Soluble Dietary Fiber

Fresh SBP was obtained from Cofco Tunhe Sugar Co., Ltd. (Changji, China). For the HD treatment, beet pulp was processed using an LC-101-0B electric blast drying oven (Hunan Lichen Instrument Technology Co., Ltd., Changsha, China). Temperature gradients of 90, 95, 100, 105, and 110 °C, along with time gradients of 40, 50, and 60 min, were assessed. For the MD treatment, an LW-60HMV tunnel microwave drying apparatus (Shandong Liwei Microwave Equipment Co., Ltd., Jinan, China) was employed, with power gradients of 4 and 6 kW and time gradients of 6, 7, 8, 9, and 10 min. Parameter optimization centers on drying efficiency as the core metric, specifically selecting the optimal conditions for both hot-air drying and microwave drying based on the shortest time required for beet pulp to reach constant weight. Preliminary findings indicated that HD at 105 °C for 50 min and MD at 6 kW for 9 min were most effective in achieving a constant sample weight, the moisture contents of the beet pulp post HD and MD treatments were 12.52 ± 1.31% and 12.36 ± 0.78%, respectively. These conditions were thus selected as optimal for subsequent investigations. The dried material was subsequently ground into powder using a mechanical crusher and sieved through a screen with a mesh size of 400 μm for further use.

The extraction of SDF was conducted in accordance with the Zhang et al. [13]. Specifically, 10 g of the sample was precisely weighed into a beaker, combined with 175 mL of 50 mmol/L maleate buffer, and homogenized using a DF-101S thermostatic magnetic stirring water bath (Youdao, Shanghai, China). Subsequently, 250 μL of α-amylase solution was introduced, and enzymatic hydrolysis was performed at 95 °C with shaking for 35 min. Following cooling to 60 °C, residues adhering to the beaker’s inner wall were collected by rinsing with 25 mL of the same buffer. Thereafter, 50 mL of dimethyl sulfoxide (DMSO) was added to facilitate starch dispersion, followed by the addition of 500 μL of protease solution and incubation at 60 °C with shaking for 30 min. Subsequently, 20 mL of 2 mol/L acetic acid solution was added, and the pH was adjusted to 4.3 using 1 mol/L NaOH or HCl. The digested mixture was transferred onto a diatomite-filled crucible pre-moistened with 3 mL of water and subjected to vacuum filtration. The residue was washed twice with 10 mL of hot water (70 °C). A 4:1 (*v*/*v*) mixture of preheated 95% ethanol (60 °C) was added to the filtrate and allowed to precipitate for 2 h at room temperature. The precipitate was collected by centrifugation at 4500 rpm for 20 min using a TDL-5M centrifuge (Sichuan Shuke Instrument Co., Ltd., Chengdu, China), completely frozen at −20 °C, and lyophilized for 48 h in the Xinyi-8A vacuum freeze dryer (Xinyi, Ningbo, China) [14]. The resulting material was ground into a fine powder using an agate mortar and stored at 4 °C until further analysis.

### 2.2. Compositional Analysis of SDF

#### 2.2.1. Molecular Weight Distribution

The molecular weight distribution was evaluated using a modified method based on the approach of Li et al. [15], employing an Agilent 1260 Infinity II GPC system (Agilent Technologies, Santa Clara, CA, USA). The mobile phase consisted of 0.1 mol/L LiCl in DMSO, delivered at a flow rate of 1.0 mL/min. Dried SDF samples (5 mg) were dissolved in 1 mL of the mobile phase solvent to achieve a concentration of 5.0 mg/mL and subsequently filtered through a PTFE syringe filter (0.45 μm) prior to injection. Separation was conducted on a DB-5ms column (30 m × 0.25 mm × 0.25 μm) maintained at 80 °C. Detection was accomplished using a refractive index detector (RID) and a multi-wavelength detector (MWD).

#### 2.2.2. Monosaccharide Composition

The monosaccharide composition was analyzed utilizing a modified protocol from Chen et al. [16], employing an Agilent 7000d triple quadrupole GC-MS system (Agilent Technologies, Santa Clara, CA, USA). The sample was dissolved in DMSO, filtered through a 0.22 μm membrane, and injected in split mode (10:1) with an injection volume of 1 μL. Separation was conducted on a DB-5ms column with helium as the carrier gas at a flow rate of 1.0 mL/min. The temperature program was as follows: an initial hold at 80 °C for 2 min, followed by a ramp to 180 °C at 10 °C/min, then to 280 °C at 5 °C/min, with a final hold for 10 min. Mass detection was performed using electron impact (EI) ionization at 230 °C, with a scan range of 35–550 *m*/*z*. Peak identification was achieved by comparison with the NIST mass spectral library.

#### 2.2.3. Fourier Transform Infrared Spectroscopy

FT-IR spectroscopy was conducted in accordance with the methodology described by Gan et al. [17], employing a Nicolet iS50 spectrometer (Thermo Fisher Scientific, Waltham, MA, USA). The sample preparation involved mixing approximately 10 mg of SDF with 100 mg of spectroscopic-grade potassium bromide (KBr). This mixture was then ground and compressed into pellets under a pressure of 20 MPa for 1 min. Spectral data were acquired in attenuated total reflectance (ATR) mode, covering the range of 4000–500 cm^−1^, with a resolution of 4 cm^−1^ and 32 scans accumulated. Background correction was applied prior to each measurement.

### 2.3. Structural Analysis of SDF

#### 2.3.1. Scanning Electron Microscopy

The microstructural morphology was investigated using a modified methodology based on Hou et al. [18]. The sample was dispersed in anhydrous ethanol via ultrasonication for 20 min, subsequently deposited on conductive carbon tape, dried, and sputter-coated with gold. Observations were conducted using a Sigma 360 scanning electron microscope (Zeiss, Oberkochen, Germany) at an accelerating voltage of 5 kV. Images were acquired at magnifications ranging from 100× to 10,000×.

#### 2.3.2. Zeta Potential

The zeta potential was assessed using a modified approach based on Liu et al. [19], utilizing a Zetasizer Nano ZS90 analyzer (Malvern Panalytical, Malvern, UK). The dried SDF was milled and sieved through an 80-mesh screen. Subsequently, 10 mg of the resulting powder was dispersed in a 1 mmol/L KCl solution to form a suspension with a concentration ranging from 0.01% to 0.1% (*w*/*v*). The suspension was subjected to ultrasonication in an ice-water bath for 15 min to ensure homogeneity. The supernatant was then transferred into a disposable capillary cell for measurement at 25 °C. Measurements were performed using a combination of dynamic light scattering (DLS) and phase analysis light scattering (M3-PALS) at a detection angle of 90° with a 633 nm laser.

### 2.4. Physicochemical Properties of SDF

#### 2.4.1. Water Holding Capacity and Swelling Capacity

The WHC and SWC were evaluated using modified methodologies as outlined by Yang et al. [20] and Xiong et al. [21]. Briefly, 5 mL of SDF was introduced into a pre-weighed centrifuge tube, followed by the addition of an excess of distilled water. The mixture was subjected to vortexing for 1 h and subsequently allowed to stand for 30 min. After centrifugation, the supernatant was discarded, and the hydrated residue was carefully blotted to remove surface water. The weight and volume of the hydrated sample were then recorded. WHC (g/g) and SWC (mL/g) were calculated using Equations (1) and (2), respectively:

(1) $$\mathrm{WHC}\;(g/g)\;=\;\frac{m_{1}-m_{0}}{m_{0}}$$

*m*_1_: Sample weight after water absorption, g; *m*_0_: Sample weight before water absorption, g.

(2) $$\mathrm{SWC}\;(\mathrm{mL}/g)=\frac{vw-vd}{m_{0}}$$

*v*_w_: Volume of the sample after absorbing water, mL; *v*_d_: Sample volume before water absorption, mL; *m*_0_: Sample weight before water absorption, g.

#### 2.4.2. Oil Holding Capacity

The OHC was evaluated by introducing 0.5 g of SDF into a centrifuge tube, followed by the addition of an excess amount of peanut oil, and thoroughly vortexing the mixture. The sample was then incubated for 1 h to ensure complete absorption of the oil. Following centrifugation, the supernatant was removed, and the residue was blotted to eliminate any unbound oil. The OHC was calculated using Equation (3):

(3) $$\mathrm{OHC}\;(g/g)\;=\;\frac{M_{1}-M_{0}}{M_{0}}$$

*M*_1_: Sample weight after oil absorption, g; *M*_0_: Sample weight before oil absorption, g.

### 2.5. Statistical Analysis

All experiments were conducted in triplicate, and the data are presented as mean values. A one-way analysis of variance (ANOVA) was performed using SPSS software 22.0 (International Business Machines Corporation, New York, NY, USA), with the significance threshold set at *p* < 0.05. Zeta potential values were calculated according to the Smoluchowski model and are reported as the average of three consecutive measurements. Molecular weight and polydispersity index were determined by GPC relative to polystyrene standards. All spectral data were processed using the software (Agilent GPC/SEC software) provided by the manufacturer.

## 3. Results and Discussion

### 3.1. Composition of SDF

#### 3.1.1. Molecular Weight Characteristics

**Figure 1:**
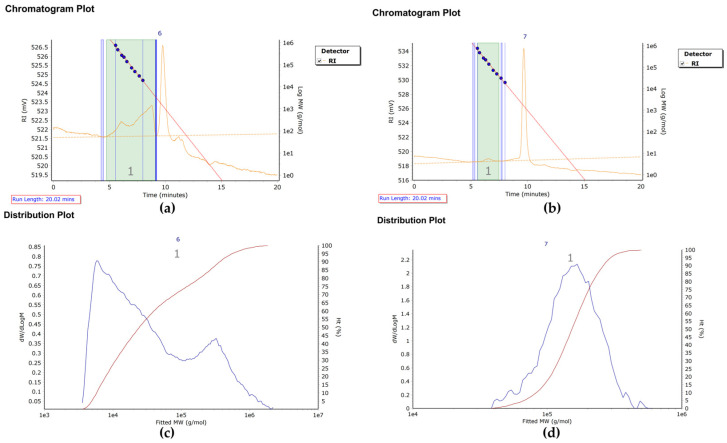
GPC and molecular weight distribution profiles of hot air drying (HD) and microwave drying (MD) of soluble dietary fiber. (**a**) HD-GPC; (**b**) MD-GPC; (**c**) HD molecular weight distribution; (**d**) MD molecular weight distribution.

GPC was employed to perform a comparative analysis of the molecular weight characteristics of polysaccharides extracted from SBP subjected to HD and MD. The number-average molecular weight (M_n_), weight-average molecular weight (Mw), and polydispersity index (PDI = Mw/M_n_) were determined to evaluate molecular weight distribution profiles [22]. As illustrated in Figure 1a,c, the HD sample exhibited values of M_n_ = 15,403 g/mol, Mw = 123,049 g/mol, and PDI = 7.989. The elevated PDI indicates a broad molecular weight distribution, suggesting the presence of both high molecular weight constituents and a minor fraction of very high-molecular-weight polymers [23]. In contrast, the chromatogram of the MD sample (Figure 1b,d) demonstrated a delayed elution profile, with M_n_ = 136,108 g/mol, Mw = 166,264 g/mol, and PDI = 1.222. The narrow molecular weight distribution and near-monodispersity (PDI = 1.222) suggest enhanced compositional homogeneity, attributable to the distinct heat and mass transfer mechanisms inherent to the two drying techniques [15]. These observations are consistent with findings reported by Tang et al. [24] on SDF from Coix seed bran, where HD induced random scission and re-aggregation of polysaccharide chains, resulting in broad molecular weight distributions (PDI > 5). Furthermore, the present results demonstrate that MD treatment significantly reduced the weight-average molecular weight, whereas HD preserved higher molecular weight fractions. This trend aligns with reports by Gan et al. [17] on microwave-assisted extraction of SDF from pomelo peel, which showed a 47% reduction in Mw and a 49% decrease in PDI, underscoring the efficient depolymerization capability of microwave irradiation. It is noteworthy that these results contrast with those documented by Wang et al. [25] for peanut shell SDF, potentially due to the heightened sensitivity of SBP components to microwave energy, leading to more pronounced depolymerization. Consequently, extrapolation of these findings to other feedstocks should be approached with caution.

#### 3.1.2. Monosaccharide Composition Analysis

**Figure 2:**
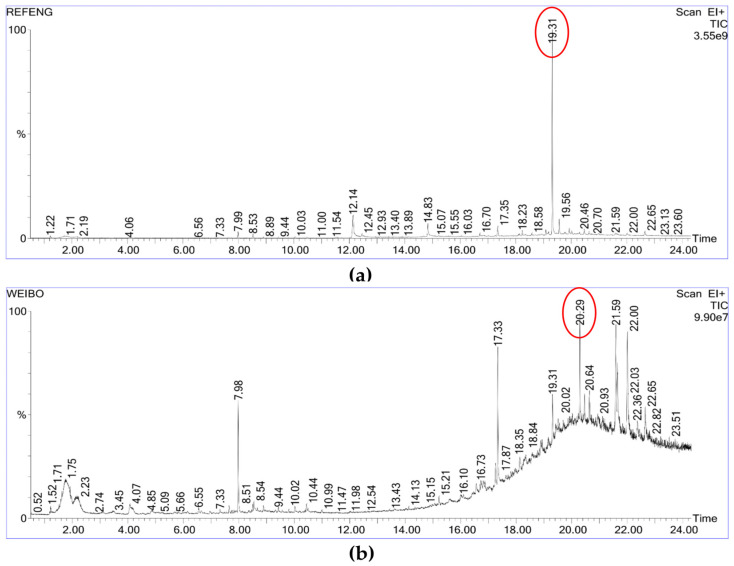
GC-MS chromatograms of hot air (**a**) and microwave (**b**) drying of soluble dietary fiber.

Volatile and derivatized compounds in HD- and MD-treated SDF from SBP were characterized using GC-MS. The resulting chromatograms are presented in Figure 2, and the major identified compounds are summarized in Table 1. The HD sample exhibited characteristic markers of thermal processing: acetic acid was detected at approximately 2 min, likely resulting from the deacetylation of pectin or hemicellulose under elevated temperatures [26]. Peaks corresponding to 5-hydroxymethylfurfural (5-HMF), 3-furaldehyde, and acetic acid were observed around 4 min and 8 min, which are recognized dehydration products derived from cellulose and hemicellulose under thermal treatment [27,28]. Two prominent peaks eluting between 12 and 14 min were tentatively identified as benzoyloxypropane (12.43%) and biphenyl-4-carboxylic acid (5.94%), potentially originating from the thermal degradation of lignin-carbohydrate complexes. A notable feature was the dominant peak at 19.313 min in the HD sample, accounting for 33.28% of the total area, suggesting the formation of phenolic-polysaccharide complexes. The prolonged dehydration during HD likely facilitated condensation reactions, leading to the formation of specific dominant products. This phenomenon is consistent with reports on distillers’ grains, where HD increased phenolic complex content by 40% compared to microwave treatment, owing to extended thermal exposure promoting recombination within lignin-carbohydrate complexes [26]. In contrast, the MD sample exhibited a more complex chromatographic profile. The most conspicuous compound was squalene (6.62%), a high-value bioactive triterpene typically associated with oleaginous matrices such as olive oil and rarely reported in dietary fiber research [29]. Its presence may be indicative of microwave-specific effects. Additionally, a complex assemblage of poorly resolved compounds was observed in the 18–21 min retention time window, collectively accounting for approximately 25% of the total peak area. This may arise from microwave-induced radical-mediated reactions or molecular rearrangements [30].

#### 3.1.3. Fourier Transform Infrared Spectroscopy Analysis

**Figure 3:**
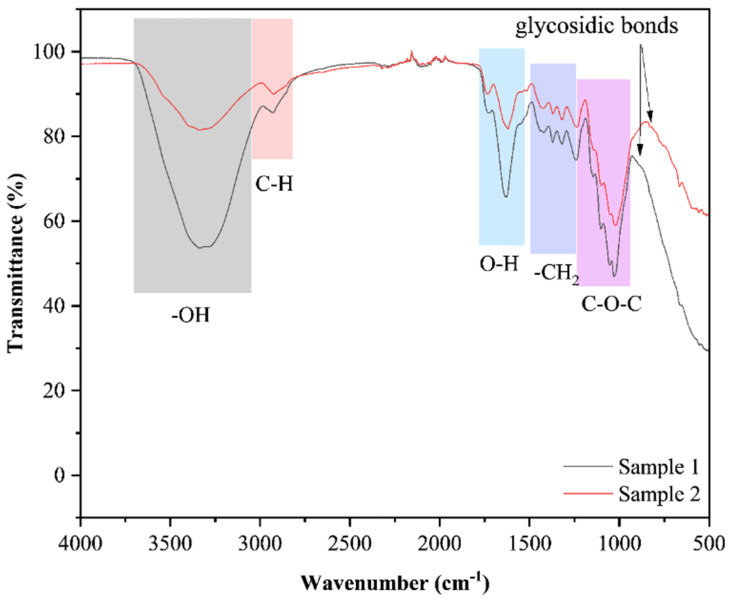
FT-IR spectra showing functional groups and glycosidic linkages in soluble dietary fiber. Sample 1 and 2 indicate hot air and microwave drying, respectively.

FT-IR spectroscopy was employed to identify functional groups in SDF samples within the spectral range of 4000–500 cm^−1^. As depicted in Figure 3, both HD and MD samples exhibited broad, intense absorption bands between 3400 and 3200 cm^−1^, corresponding to -OH and N-H stretching vibrations. The slightly broader absorption observed in the HD sample may suggest the presence of retained hydrogen-bonded water, which aligns with the extended drying duration and slower moisture migration characteristic of HD. The increased intensity of this band in HD-SDF indicates a higher abundance of surface-exposed -OH groups [31]. Minimal differences were observed between the samples in the 2850–1790 cm^−1^ region, indicating that the drying methods had limited impact on aliphatic structures. The absorption features in this region can be attributed to C-H stretching vibrations within glucose rings [32]. The band at 1640–1600 cm^−1^ may present composite signals, potentially originating from the stretching vibrations of adsorbed water O-H groups and vibrations associated with carboxylates in lignin/hemicellulose [33]. Notably, MD samples exhibit significantly stronger absorption intensity in this region. Bands between 1445 and 1220 cm^−1^ may be assigned to CH_2_ bending vibrations [13]. The most intense absorption occurred at 1160–1030 cm^−1^, attributable to asymmetric stretching of C-O-C and C-O bonds, characteristic of glycosidic linkages and carbohydrate skeletal vibrations [34]. The markedly heightened absorption intensity in MD-SDF suggests that microwave treatment induced more pronounced structural alterations in cell wall polysaccharides, potentially resulting in greater depolymerization, cleavage, or structural loosening, thereby exposing additional polar functional groups [35]. The absorption peak at 895–890 cm^−1^ is consistent with typical glycosidic bond vibrations [36].

### 3.2. Structural Characterization of SDF

#### 3.2.1. Scanning Electron Microscopy Analysis

**Figure 4:**
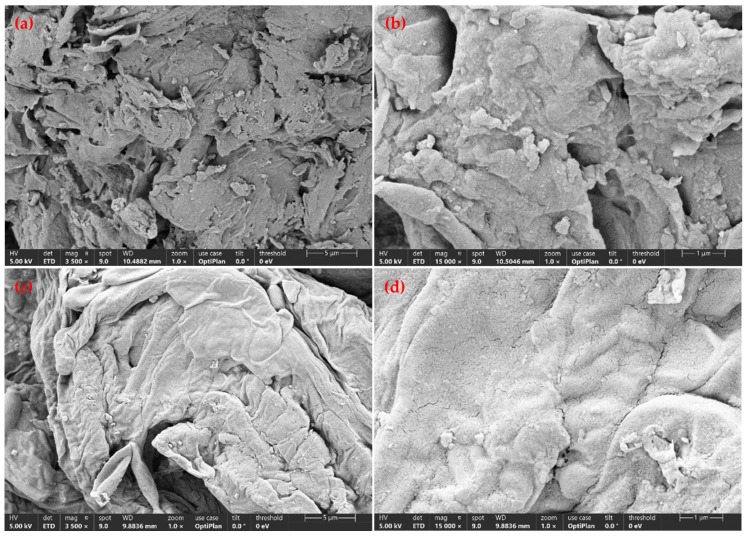
Scanning electron micrographs of hot air drying (HD) and microwave drying (MD) of soluble dietary fiber. (**a**) HD at 3500×; (**b**) HD at 15,000×; (**c**) MD at 3500×; (**d**) MD at 15,000×.

The microstructural characteristics of SDF, which significantly influence its porosity and surface properties [37], were examined using SEM. A comparative analysis of HD- and MD-SDF revealed distinct morphological differences. As depicted in Figure 4a,b, the HD sample exhibited a compacted and contracted morphology. The intrinsic cellular honeycomb or porous architecture of HD-SDF was extensively compromised and collapsed, resulting in reduced porosity [38]. During HD, thermal energy is transferred from the surface inward, causing premature surface moisture evaporation and the formation of a rigid crust. This process impedes internal moisture migration, leading to insufficient internal steam pressure and ultimately causing structural collapse and adhesion mediated by surface tension and capillary forces [39]. In contrast, the MD sample demonstrated an open, highly porous, three-dimensional structure (Figure 4c,d). The structural integrity of MD-SDF was notably preserved, with significantly reduced shrinkage compared to the HD sample. This outcome is attributed to the rapid and volumetric heating provided by microwave energy, which induces instantaneous vaporization of internal moisture. The resulting buildup of internal steam pressure exceeds the mechanical strength of cell walls, leading to rupture and the formation of an interconnected porous network [40]. This highly porous structure suggests a substantially increased specific surface area, which predicts superior rehydration performance, crisp textural attributes, and rapid dissolution [22]. Conversely, the dense morphology of HD samples may require greater masticatory effort and exhibit delayed rehydration.

#### 3.2.2. Zeta Potential Analysis

**Figure 5:**
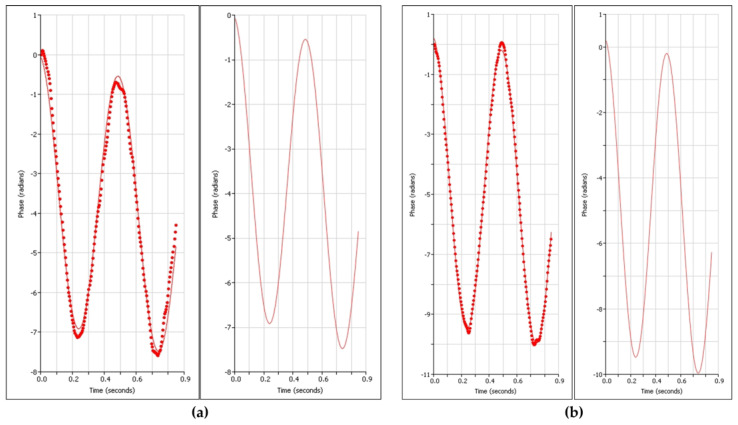
Zeta potential distribution of hot air (**a**) and microwave (**b**) drying of soluble dietary fiber.

Zeta potential was assessed to determine the surface charge characteristics and colloidal stability of SDF dispersions derived from samples subjected to various drying methods. The magnitude of the zeta potential reflects the degree of electrostatic repulsion between particles; higher absolute values indicate enhanced stability against aggregation [41]. As depicted in Figure 5, the average zeta potential of the HD sample was −18.88 mV, indicating a critically unstable dispersion system. Systems with |ζ| < 20 mV are generally considered susceptible to aggregation due to insufficient electrostatic repulsion to counteract van der Waals attractive forces [42]. Conversely, the MD sample exhibited an average zeta potential of −24.47 mV. This significantly higher value suggests that MD increased the net negative surface charge of the particles, resulting in a dispersion system of moderate stability [43]. This increase may be attributed to the unraveling of SDF molecular chains, which exposes additional ionizable carboxyl groups (-COO^−^), thereby enhancing the surface negative charge density. This interpretation is consistent with the intensified carbonyl-related absorption band observed in the FT-IR analysis of MD samples. Consequently, the physical stability and suspension behavior of microwave-dried SBP powder in aqueous media are expected to be substantially superior to those of the hot air-dried counterpart [44,45].

### 3.3. Physicochemical Functional Properties

The influence of the drying method on the functional properties of SDF, specifically WHC, OHC, and SWC were evaluated, as these properties determine its potential application in food systems as a thickening agent, stabilizer, fat substitute, and texturizer, as well as its role in modulating satiety [46]. The WHC, SWC, and OHC were calculated according to Equations (1)–(3), and the results are presented in Table 2. The WHC of the microwave-dried (MD) sample was significantly higher than that of the hot air-dried (HD) sample, indicating a significantly enhanced ability of microwave-dried material to retain water under centrifugal force [47]. In contrast, the SWC of the HD sample was significantly higher than that of the MD sample by 0.30 mL/g, suggesting that hot air-dried pulp undergoes greater volumetric expansion in aqueous media [22]. The OHC values of the two samples showed no significant difference, indicating negligible differences in oil retention. Collectively, these results suggest that MD offers a significant advantage in enhancing water retention, whereas HD results in superior swelling characteristics. Both methods produced comparable oil-binding capacities. These findings are consistent with the conclusions drawn by Sui et al. [48], indicating that microwave processing can selectively modify the functional attributes of SDF, enhancing its hydration-related properties. Therefore, microwave-dried SDF may be more suitable for applications requiring rapid hydration and high water retention, whereas hot air-dried products may be preferable in applications where high volumetric expansion and enhanced satiety are desired [17].

## 4. Conclusions

This study investigated the differential effects of HD and MD on the structural, physicochemical, and functional properties of SDF derived from SBP, attributable to their distinct heating mechanisms. The results indicated that SDF obtained through HD exhibited polysaccharide fractions with a higher molecular weight (Mw = 123,049 g/mol) and a broader molecular weight distribution (PDI = 7.989), along with the formation of thermal degradation products such as 5-hydroxymethylfurfural and lignin-derived compounds. HD also resulted in a dense and shrunken microstructure, lower zeta potential, and reduced colloidal stability, while enhancing SWC, making it suitable for applications requiring high volume expansion, such as weight-management foods. In contrast, MD produced SDF with a lower molecular weight and more homogeneous distribution (Mw = 166,264 g/mol, PDI = 1.222), retained higher levels of bioactive compounds such as squalene, and formed a loose, porous microstructure. These structural attributes contributed to a higher WHC and a greater Zeta potential (−24.47 mV), indicating improved dispersion stability in aqueous systems. These characteristics render MD-SBP an ideal raw material for functional food applications. This study underscores the importance of selecting the appropriate drying method based on desired functional properties, thereby providing a scientific foundation for optimizing the valorization of SBP. Further research focusing on parameter optimization and in vivo validation is warranted to enhance the practical applicability of these findings.

## Figures and Tables

**Table 1:** Major compounds identified by GC-MS in hot air drying of soluble dietary fiber and microwave drying of soluble dietary fiber.

Method	RT (min)	Compound Name ^1^	Match/R Match	Area (%)	CAS No.	Identification Confidence ^2^
Hot air Drying	1.775 ^3^	Ethyne, fluoro-	913/930	3.914	2971-09-9	High confidence
	2.154	Aceticacid	912/927	1.686	64-19-7	High confidence
	4.073	3-Furaldehyde	916/924	0.417	498-60-2	High confidence
	8.526	5-Hydroxymethylfurfural	915/918	0.784	67-47-0	High confidence
	12.137	Benzoicacid,2-(1-oxopropyl)-	813/822	12.429	2360-45-4	Tentative
	14.831	Biphenyl-4-carboxylicacid	897/908	5.936	92-92-2	Tentative
	17.349	1,8-Diazacyclotetradecane-2,9-dione	870/873	2.254	5776-79-4	High confidence
	19.313	2H-pyrrol-2-one,1,5-dihydro-4-hydroxy-3-[(4-methylphenyl)thio]-1-phenyl-	619/644	33.279	/	Unidentified
Microwave Drying	1.775 ^3^	Ethyne, fluoro-	920/949	4.977	2713-09-9	High confidence
	2.176	Aceticacid	813/907	2.668	64-19-7	High confidence
	17.332	1,8-Diazacyclotetradecane-2,9-dione	862/867	1.941	5776-79-4	High confidence
	20.293	Squalene	783/833	6.620	111-02-4	High confidence
	20.639	(R)-9-[(R)-2-(Hydroxymethyl)pyrrolidin-1-yl]-3-isobutyl-3,4-dihydro-2H-benzo[b][1,4,5]oxathiazepine1,1-dioxide	569/629	5.362	1334427-03-0	Tentative
	21.59	Furanic/aromatic pyrolysis product	540/580	3.29	/	Tentative
	22.003	2,2,4-Trimethyl-4-(4-hydroxyphenyl)chroman,tert-butyldimethylsilylether	517/533	3.295	/	Tentative
	18–21	Complex unknown product group ^4^	400–550	≈25	/	Unidentified

^1^ Compound identification was conducted by comparing the results with the NIST mass spectral library and retention indices (RI). ^2^ The confidence in identification was categorized as follows: High confidence was assigned when both match and reverse match factors > 800, or when supported by RI; Tentative identification was proposed based on the best spectral match despite low similarity; Unidentified compounds, which exhibited low spectral similarity, were reported as valuable product groups. ^3^ The peak observed at RT 1.775 min was identified as a residual derivatization reagent. ^4^ The term “Complex unknown mixture” refers to compounds with low spectral similarity (match factor 400–550), which are likely products of the Maillard reaction and pyrolysis unique to MD.

**Table 2:** Physicochemical properties of hot air and microwave drying of soluble dietary fiber.

Method	Water Holding Capacity (g/g)	Swelling Capacity (mL/g)	Oil Holding Capacity (g/g)
Hot air Drying	4.71 ± 0.08 ^a^	5.18 ± 0.05 ^a^	2.59 ± 0.07 ^a^
Microwave Drying	5.01 ± 0.05 ^b^	4.88 ± 0.14 ^b^	2.61 ± 0.07 ^a^

Values are expressed as mean ± standard deviation (*n* = 3). Different lowercase superscript letters within the same column indicate significant differences (*p* < 0.05).

## Data Availability

The original contributions presented in this study are included in the article. Further inquiries can be directed to the corresponding author.
